# The (un)caring experienced by racialized and/or ethnoculturally diverse residents in supportive living: a qualitative study

**DOI:** 10.1186/s12877-023-04636-0

**Published:** 2024-01-20

**Authors:** Stephanie A. Chamberlain, Jordana Salma, Hongmei Tong, Savera, Jingfeng Wu, Andrea Gruneir

**Affiliations:** 1 Faculty of Nursing, College of Health Sciences, University of Alberta, Edmonton, AB Canada; 2 Faculty of Health and Community Studies, MacEwan University, Edmonton, Alberta Canada; 3 Engineering Education Research, College of Engineering, University of Michigan, Ann Arbor, MI USA; 4 Department of Family Medicine, College of Health Sciences, University of Alberta, Edmonton, Alberta Canada

**Keywords:** Supportive living, Culturally appropriate care, Diverse residents

## Abstract

**Background:**

Racialized and/or ethnocultural minority older adults in supportive living settings may not have access to appropriate services and activities. Most supportive living facilities are mainstream (not specific to one group); however, culturally specific facilities are purpose-built to accommodate older adults from a particular group. Our objective was to describe the perspectives of diverse participants about access to culturally appropriate care, accessible services, and social and recreation activities in culturally specific and mainstream (non-specific) supportive living facilities.

**Methods:**

We conducted semi-structured interviews with 21 people (11 staff, 8 family members, 2 residents) from 7 supportive living homes (2 culturally specific and 5 mainstream) in Alberta, Canada. We used a rapid qualitative inquiry approach to structure the data collection and analysis.

**Results:**

Staff and family members described challenges in accessing culturally appropriate care in mainstream facilities. Family members expressed guilt and shame when their relative moved to supportive living, and they specifically described long waitlists for beds in culturally specific homes. Once in the facility, language barriers contributed to quality of care issues (e.g., delayed assessments) and challenges accessing recreation and social activities in both mainstream and culturally specific homes. Mainstream facilities often did not have appropriate food options and had limited supports for religious practices. Residents who had better English language proficiency had an easier transition to supportive living.

**Conclusions:**

Racialized and/or ethnoculturally diverse residents in mainstream supportive living facilities did not receive culturally appropriate care. Creating standalone facilities for every cultural group is not feasible; therefore, we must improve the care in mainstream facilities, including recruiting more diverse staff and integrating a wider range of recreation and religious services and food options.

**Supplementary Information:**

The online version contains supplementary material available at 10.1186/s12877-023-04636-0.

## Background

Many high-income countries, like the US and Canada, have aging populations and the use of residential care settings (e.g., supportive [assisted] living and nursing homes) for older adults is common [1]. These countries also experience high rates of immigration, including from countries where the use of care settings is far less common [2]. Many of these immigrant groups are racialized, a term to denote persons of colour and visible minority populations [3]. Understanding the experience of racialized, immigrant, and/or ethnocultural minority residents (hereafter described as racialized and ethnocultural minority residents) in residential settings is critical because the number of residents from this group is increasing, and compared with the general population, these residents are at greater risk of poor health outcomes [4, 5] and poor quality of life [6, 7]. Systemic barriers contributing to poor health and quality of life include a lack of religious, cultural, or linguistically congruent services [8–10]. Culturally specific care homes exist to accommodate older adults from certain racial and/or ethnocultural backgrounds; these are purpose-built facilities that cater to residents who are predominantly from a particular group, with staff who speak the appropriate language and customized food options and cultural and/or religious activities [11, 12]. At a national level, the number of culturally or ethnically specific homes is not known. In Ontario, Canada’s most populous province, approximately 9% (56/624) of nursing homes (NH) are culturally specific [13]. In Alberta, 3% (5/163) of supportive living (SL) homes are culturally specific. Unfortunately, the small number of culturally specific NH or SL homes in Canada have long wait times for entry. Access to continuing care facilities in Canada is through a single point of entry, where the person’s needs are assessed to determine their entry to a specific level of care. Individuals can indicate their preferences for facilities like culturally specific homes, and those on the waitlist are prioritized for a bed based on their current care needs and urgency of placement. A report from the Wellesley Institute found that those who applied to live in a culturally specific home waited approximately six months longer than those who applied to a mainstream (non-culturally specific) home [14]. A recent study from Ontario, Canada compared the time to placement in a NH between recent immigrants and long-standing residents. Significantly longer wait times were experienced by recent immigrants applying to a culturally specific home versus a mainstream home (425 days waiting versus 162 days waiting) [15]. Our project examined the decision-making process and transition to living in culturally specific or mainstream SL facilities for racialized and culturally diverse older adult and their family members.

Most SL facilities are not culturally specific. Racialized and ethnocultural minority residents in mainstream homes experience issues related to quality of care and quality of life, including high levels of loneliness [16]. Older adults, particularly those who migrated later in life and have challenges speaking English, face significant barriers to participating in activities and accessing social supports [17]. A recent scoping review of immigrant and refugee older adults found that loneliness and social isolation may be exacerbated for racialized and ethnocultural minority older adults because of diminished access to external social networks, lack of available or culturally appropriate activities, and language barriers [18]. However, that scoping review only examined research in the community and did not examine older adults in SL or NH. Understanding the experience of racialized and immigrant residents in care settings, particularly as it relates to loneliness and participation in social activities, is essential to identify potentially modifiable barriers contributing to poor quality of life for these older adults. The purpose of this study was to describe the perspectives of diverse stakeholders in SL about access to culturally appropriate care, accessible services, and social and recreation activities for South Asian, Chinese, and Arab communities.

## Methods

### Design

This project used a rapid qualitative inquiry (RQI) methodology to guide the data collection and analysis [19]. RQI is an as intensive, team-based qualitative approach with a focus on the insider’s (emic) perspective and iterative data analysis to quickly develop a preliminary understanding of the situation. RQI is used when a project needs to be completed within a constrained timeframe and it involves specific and targeted research questions. The techniques are action-oriented and focus on using the results to inform other work. An RQI method was appropriate given our constrained project timeframe (one year total, including ethics submission, hiring staff, recruitment, data collection, and analysis) and our targeted research questions. Data were collected using semi-structured interview methods. We conducted interviews with residents, family members, and staff from SL homes in Alberta, Canada, guided by an intersectional framework. Intersectionality posits that social constructions such as race, class, gender, ability, age, and sexual orientation create interacting and interdependent systems of discrimination and oppression [20]. Race, ethnicity, cultural identity, immigration status, and religious affiliation were core to our sampling. Along these lines, it is important to recognize that most older adults in Canada from our target communities are first generation immigrants and so often being racialized and an immigrant intersect in specific ways for this population [21]. We acknowledge that not all immigrants are racialized minorities and not all racialized communities are immigrants, and these groups may have different experiences and needs [22]. Our interview guides collected these personal characteristics and included research questions related to participant sociodemographic characteristics and access to appropriate services and activities.

### Setting and sample

This project was conducted from January 2021 to June 2021 in SL homes in Alberta, Canada. Similar to assisted living in the United States, SL is situated between community-based home care and NH care. SL aims to provide some care and services in a communal setting while enabling older adults to maintain some independence and autonomy [23, 24]. Older adults and their families want these settings to provide a ‘homelike’ alternative where family support is still needed alongside some formal care and supervision. We focused on SL because it is the fastest-growing sector of the publicly funded continuing care system in Alberta, yet these settings have yet to be studied as extensively as NH and there are numerous concerns about their ability to provide quality care for residents. What little research there is on SL in Canada has found that SL residents are similar to NH residents in terms of age, cognitive impairment, chronic conditions, and functional limitations, yet SL facilities have fewer services and less staffing resources lack the staffing resources of NHs [23, 25–29]. Recent studies comparing NH and SL in Alberta before and during the COVID-19 pandemic found troubling differences in the settings, including higher rates of antipsychotic use, pain, and depressive symptoms in SL compared to NHs [30]. A recent literature review examined the needs of older people with dementia from culturally and linguistically diverse backgrounds but none of the included studies specifically examined residents in SL facilities [31]. This demonstrates a gap in what is known about the quality of care and quality of life of SL residents that our work aims to address.

Our population of interest was racialized and ethnocultural minority SL residents and family members. We specifically focused on SL residents and family from the South Asian, Chinese, and Arab communities, which represent the largest population of immigrants in Alberta; SL homes have been established by these communities in the province. Culturally specific homes were created in Alberta through partnerships between community-based organizations and provincial funding initiatives; these homes are intended to provide more culturally appropriate care to older adults from these communities. Entry to culturally specific homes in Alberta is through the same process as mainstream homes, where individuals are referred to a placement office where a needs assessment and admission to care is coordinated (if needed). If assessed for facility-based care, individuals can choose at least one preferred site. Each site has a different waitlist depending on occupancy and demand.

### Recruitment

Our study included residents, family members, and SL staff. We used convenience sampling methods given that the research focused on a new area of inquiry (culturally appropriate care in SL homes) [32]. Inclusion criteria for residents was being from the South Asian, Chinese, or Arab communities. The inclusion criteria for family participants was having a family member from one of the target communities currently living in a licensed SL home in Alberta. Staff participants had to be currently working in a licensed SL home in Alberta. Staff could be from any racial or ethnocultural background but needed to have experience working with resident and family groups from the targeted communities.

We recruited participants from 7 SL homes (2 culturally specific and 5 mainstream). Homes were situated in the two largest health regions in Alberta (Calgary, Edmonton), and were identified through publicly available lists. Culturally specific homes catered to our target groups (Chinese, Arab). SL administrators or their equivalent received a recruitment email describing the purpose and objectives of the study. Attached to the email was a one-page study description with our contact information. Administrators distributed the recruitment email to other staff and family via newsletters and email listservs. Potential staff and family participants contacted the study team directly by email or telephone to schedule an interview. Staff reached out to residents directly with study information, and residents contacted study staff by phone or had a staff member facilitate contact, if needed.

We recruited family members by advertising the study through relevant community groups (e.g., Seniors Association of Greater Alberta, Multicultural Health Brokers, CoMag [Muslims Aging Gracefully], African Center, SHAAMA Center, and AGE of Wisdom-Edmonton Seniors Coordinating Council).

### Team

RQI is defined by its team-based, insider perspective. Intensive teamwork is a substitute for the prolonged fieldwork of most qualitative research approaches. Peer research assistants and team members from the communities of interest are included to foster an insider perspective. We hired two peer research assistants (S & JW) from existing community-based connections within the target communities to assist in recruitment, interviewing, and analysis. Both peer research assistants had previously participated as academic interviewers with the target communities. The team lead (SC) who has experience in qualitative interviewing led their training on interview techniques and engaging with older adults in SL settings. SC led the initial interviews, sat in on all the other interviews, and oversaw all the analyses.

Once a potential interview participant provided their approval to be contacted, the research team lead (SC) determined if the participant met the inclusion criteria and inquired about their ethnocultural identity and any language considerations, then assigned a peer research assistant to the eligible participant. Our interviews were conducted in English by participant request because they felt comfortable conversing in English. However, research assistants were present if clarification was needed in the participant’s other spoken language. The diverse research team included those with expertise in gerontology and immigrant health. Some of the research team members identified as members of the target communities and spoke the languages of participants. This supported the credibility of the RQI process, which emphasizes the insider perspective to understand and contextualize participant experience.

### Interview guides

Rapid qualitative techniques typically rely on semi-structured data collection that provides an overall structure to the concepts in question, but allows people space to provide their own experience by using open-ended questions (see Supplementary file 1 for copies of the interview guides). The interviews focused on topics such as the availability and access to culturally appropriate social programs, opportunities for maintaining connection to family and friends, loneliness, family roles and tasks, and any opportunities or challenges with accessing support in a language other than English. The interview guides were developed collaboratively by the team members. The guides also contained closed-ended questions to capture participant demographic characteristics.

### Data collection

Interviews were conducted and recorded using the Zoom platform, a collaborative, cloud-based videoconferencing service offering online meetings, group messaging services, and secure recording of sessions (Zoom Video Communications Inc., 2016). All recordings were stored securely on local drives and later transcribed. All transcripts were shared with the participants so that they could provide corrections to facts and their own interpretation of the situation. One participant provided more details after reviewing their transcript; however, no changes to the transcripts were requested by this participant or others. While there were only two residents, we retained them in the study because the data derived from these participants does not contradict what was said by the other participants. We examined our study participants as a collective and all participants shared similar patterns of core experiences and perspectives in SL on the topic of study. Data collection was suspended when informational redundancy was achieved (i.e., new interviews did not result in new themes or sub-themes) [33].

### Analysis

Data analysis occurred concurrently with data collection. Before beginning data collection, we used our semi-structured interview guide questions to identify the potential primary themes. Once we began data collection, these themes were examined and refined based on our participant responses. Below we describe the analytic steps that we undertook and that follow the RQI approach.

1. We created a summary template for each individual interview. The summary templates are structured based on column names (initial themes) that correspond with each interview question. Templates were populated after each individual interview.
2. Two team members (S, JW) independently reviewed the interview transcript and completed the summary sheet. For each interview, the summary templates were reviewed by an investigator (SC) to determine if there should be changes or additions. Summaries had space for unexpected findings that were not in the interview guides. A team member reviewed each of the independently completed summary sheets and consolidated them into one final interview summary. Discrepancies between the two sheets were discussed by the team and resolved by consensus.
3. Completed summaries were then transferred to a matrix. The matrix had respondents in rows and the themes in columns (see Supplementary file 2 for an example data matrix template).
4. Once the matrix was completed, we developed a codebook that described the depth and breadth of the data in each theme and sub-theme (as needed).

In the RQI method, the concept of triangulation is focused on intensive teamwork and the use of multiple perspectives to discern the meaning of results [19]. In RQI, successful triangulation requires a team of selected members who bring different theoretical, disciplinary, and methodological backgrounds to a shared project. In our study, the diverse team membership also resulted in theoretical triangulation where multiple academic perspectives including gerontology, nursing, epidemiology, health services, sociology, and social work were used to interpret the data. Research areas ranged from applied health services research and quantitative methodologies to cross-cultural and transnational dimensions of aging. In addition to our semi-structured interviews, our analysis included the use of comprehensive note taking and audit trails during the data collection and analysis phase.

## Results

We interviewed 21 people (11 staff, 8 family members, 2 residents) (Table 1). Of the 11 staff, 6 worked in culturally specific homes and 5 worked in mainstream facilities. Most staff were women, born outside of Canada, and spoke at least one language other than English (e.g., Urdu, Hindi, Mandarin). Family member participants were children or married to children of SL residents, over half were women, and all spoke at least one language other than English. Both residents lived in a culturally specific home. Our analysis resulted in 5 primary themes and 8 associated sub-themes.

**Table 1:** Participant characteristics

	**N (%)**
**RESIDENTS (*N*=2)**	
**Type of home**	
Culturally specific	2 (100)
**Gender**	
Woman	1 (50)
Man	1 (50)
**Spoke a language in addition to English**	2 (100)
**FAMILY MEMBERS (*N*=8)**	
**Type of home**	
Culturally specific	4 (50)
Mainstream	4 (50)
**Gender**	
Woman	5 (62.5)
Man	3 (37.5)
**Spoke a language in addition to English**	8 (100)
**STAFF MEMBERS (*N*=11)**	
**Type of home**	
Mainstream	5 (45.5)
Culturally specific	6 (54.5)
**Gender**	
Woman	10 (90.9)
Man	1 (9.1)
**Spoke a language in addition to English**	9 (81.8)
**Staff roles**	
Licensed practical nurse	4 (36.4)
Health care aide	3 (27.3)
Director of care/Manager	2 (18.2)
Clinical Educator/Unit Clerk	2 (18.2)

### Spectrum of family caregiving

#### Transition to SL

Family members and residents described the period before they moved to the SL home, including their previous care needs and the steps that led them to need SL. Before moving to SL, family members described their relative’s health progressively declining and how they experienced challenges accessing sufficient home care services. In most cases, residents had received the maximum amount of available publicly funded home care services and were advised to move to a care setting. Family members described delaying the move if possible, citing cultural expectations to care for loved ones at home. In most cases, residents had been in and out of hospital, and following a critical health event they were moved directly from hospital to SL.

I’m also in my 50s and my husband works overseas, we never wanted to send [her] it’s our culture…always the parents are with us, especially with the son…but it was getting harder and harder. We tried keeping a nurse at home, like what the government gives, but it didn’t work for us and it didn’t work for her then she was again hospitalized and they said it’s better to move her. (Family, mainstream home)

Families were heavily involved in selecting potential SL homes. Family members with relatives in mainstream homes described visiting multiple sites, often choosing the one that was closest in geographic proximity to their home to facilitate frequent visits. Family members of residents moving to mainstream homes described looking for staff that appeared to be from similar racial or ethnocultural backgrounds and listening for staff or residents that spoke the same language as their family member. For example, “I visited a place…it was really very nice, high-end… but for my mother-in-law… it just wasn’t good because I didn’t see any Indian or anybody who she can talk to” (Family, mainstream home). Family typically had to select a mainstream home because a culturally specific facility was not available or the need for immediate placement was too great. Residents and family members who had culturally specific facility options described being on the wait list for their preferred home years in advance. Families of residents in culturally specific homes opted to wait for a bed and care for their loved one in the community because they wanted their relative to live in a facility with familiar language, religious practices, and food. One participant said, “They can do their evening prayers as a community, that’s what attracted her the most” (Family, culturally specific home).

#### Shame and guilt

Staff described the family members of residents from diverse ethnocultural backgrounds as involved, attentive, and frequent visitors. Family would visit residents daily or multiple times per week. During the COVID-19 visitor ban, most family members adapted with outdoor visits, window visits, or video/phone calls. Family of residents in mainstream homes would often bring in familiar food for the resident.

Family members of relatives in mainstream homes described intense feelings of guilt, depression, and shame over their loved one moving to the SL facility. They described how it was an uncommon practice in their culture and found the decision-making process emotionally taxing and painful. One daughter described her intense guilt after her father moved to SL: “We still haven’t gotten over it, not me and not my brothers and sisters, because we felt that we had abandoned my father” (Family, mainstream home).

Conversely, family members of residents in culturally specific homes did not express such negative emotions about the transition to SL. Many described feeling positive about the move, because they often knew other family members and residents in the facility from their community and felt that their relative was surrounded by familiar community. These family members considered moving to the culturally specific home as an ideal option.

### Familiar activities and food

Family, residents, and staff described the disparate access, availability, and appropriateness of recreation and social activities in SL homes. In general, mainstream facilities only celebrated Christian-based holidays (e.g., Christmas, Easter). “The traditional/Western homes, all the activities were very related to White people. He [resident] had no idea how to play Bingo, he’d never done it in his own life” (Family, mainstream home)

#### Recreation and social activities

Culturally specific homes celebrated both Christian holidays and other cultural festivals. In these homes, cultural and religious activities (i.e., evening prayers, traditional festival celebrations) were offered in multiple languages. Local community groups, religious leaders, and volunteer groups were present and involved in organizing activities and events in culturally specific homes.

There were several issues related to participation in recreation and social activities in mainstream homes. Barriers included language issues (residents unable to speak English) and having no staff from the resident’s culture to facilitate non-Christian activities. Given the comparatively small number of residents from racial or ethnoculturally diverse backgrounds in mainstream homes, staff felt it was challenging to dedicate already limited recreation resources to these residents. Staff members in mainstream facilities described limited external volunteer supports and cited a need to engage family members or members from the different cultural communities to lead activities and celebrations in the facility.

Here we have nothing specific, it’s just basic recreation stuff like typical Canadian you know…they’ll have arts and crafts, but nothing really to commemorate the different cultures. We didn’t even celebrate Black History Month…. no Chinese New Year, it’s not very culturally sensitive environment for the residents. (Staff, mainstream home)

#### Familiar food

Family members of residents in mainstream homes described challenges accessing religious or culturally familiar food. Muslim family members described their distress that the homes did not offer any Halal food options, and the residents only option was to forgo their religious requirements or adopt a vegetarian diet.

No, we don’t even have heart healthy diet, let alone, you know Halal and Kosher… they don’t have any access to that. They’re getting our typical Canadian food…it’s not an option for religious practices. (Staff, mainstream home)

In some cases, staff attempted to coordinate with the kitchen to adapt to the dietary restrictions, but family members said it was often not successful. In the culturally specific homes, they offered both Eastern and Western menus. “They serve Chinese food, and my mom can eat both…my mom is quite assimilated that way she can eat any kind of food, but still Chinese food is her main staple” (Family, culturally specific home). Residents in the culturally specific homes appreciated having the options for both styles of food. One resident remarked while he appreciated the food options, his country contained many different food cultures and regional cuisines and noted it would always be challenging for SL facilities to make food that was familiar and satisfied everyone, even in a culturally specific home.

### Loneliness

All participants (residents, family, staff) were asked to describe residents’ experience of loneliness. Loneliness was prevalent in both types of homes; however, language barriers and having reduced ability to communicate with staff and other residents compounded feelings of loneliness for residents in mainstream homes. “To me I think she’s very lonely because she said I don’t have anyone to talk to you and even though she speaks the language people around her are not of her caliber of mental state” (Family, culturally specific home). Residents living in mainstream homes were described by staff and family as extremely at risk of loneliness, primarily because of language barriers. Loneliness and language were closely linked in our findings. Although family and residents in the culturally specific homes did indicate that residents also experienced loneliness, at least those residents could talk to staff and other residents in their own language. Their language or cultural background was not the predominant barrier to social participation.

The food they can probably handle, but the English language is definitely a big barrier for a lot of people, they are isolated or depressed because they can’t talk. You’re with the people and yet you cannot communicate with them, you’re alone because you cannot voice out your opinion and your feelings. (Staff, mainstream home)

Residents of mainstream facilities did not have other residents or staff who spoke their language. Loneliness was particularly prevalent during COVID-19 visitor restrictions when the only time these residents could verbally communicate with someone was during twice weekly Zoom calls with their family.

My father felt it [the loneliness] more acutely because he was like a fish out of water in such a White facility. Everybody else I’m sure felt lonesome and neglected too, but at least they had each other, they had the language, they had the culture. (Family, mainstream home)

### Language and care

Family and staff in both mainstream and culturally specific homes and the residents in culturally specific homes described significant challenges due to language barriers; however, the issues were more prevalent in mainstream facilities where there was often no other person in the facility available to translate for or communicate with the resident. Clinical staff (licensed practical nurses) described their concern over language barriers because it impeded their ability to complete accurate resident assessments. They described times when residents had fallen or appeared to be in pain, and staff were unable to obtain information about the incident, such as the location or duration of the pain. “One of our residents, a Cantonese lady, she fell and we called the son…because we didn’t know if it was broke, if she’s in any pain” (Staff, mainstream home). Another said “We end up calling her [the family member] to interpret what she’s [resident] trying to tell us and then we found out she’s complaining about dizziness or blurry vision or … headache, it’s hard” (Staff, mainstream home).

In the culturally specific homes, there were still instances when staff did not speak the specific language or dialect of a resident, but there were often staff elsewhere on site who could translate. Having staff available on-site, particularly because SL facilities do not have on-site interpretation services was important to providing high quality clinical care and resident quality of life.

#### Strategies and approaches

Participants in mainstream and culturally specific homes described a number of strategies that were used by staff to communicate with residents who did not speak the same language. Staff in mainstream homes who could not communicate with the resident noted that they relied on hand signals, body language, word or picture boards, and the Google Translate app on their phones. Staff in mainstream homes also called family members to act as an intermediary when they needed information from the resident, and requested that family write down frequently used words or phrases in the resident’s language for the staff.

We had one instance where one of our residents [who was Cantonese], she fell and we called the son and the son is great, just so that they could ask her about moving her leg certain way, because we didn’t know if it was broke, if she's in any pain and stuff like that. We had to call him so now we have a couple of more words written down so that staff can kind of ask her certain things to add to the board, because there were a few words we didn’t have. (Staff, mainstream home)

Challenges communicating with residents often led to conflict and frustration for the residents and staff. However, when a staff member was able to communicate with the resident, this could ease agitation and improve the resident’s mood.

I remember one of our residents, she speaks the same language as our [my] dialect and she’s always agitated. When I come approach her with the same dialect I can see her reaction, she calms down, and you can see that she’s so happy to hear that dialect so it’s definitely helpful to have different backgrounds of people working in a facility. (Staff, mainstream home)

### Culturally competent care

Understanding innate or overt cultural practices was one reason that residents and family gravitated to culturally specific facilities. For example, family and residents in a Chinese SL home described how the facility always had warm tea and beverages available, as was customary in many Chinese homes. When family members had relatives in mainstream homes, this practice was not offered or understood. A family member from a culturally specific home said that these subtle differences were more apparent to her because she had one parent living in a mainstream home and the other in a cultural home. The culturally specific home did not have pet therapy, consistent with cultural beliefs around animals and cleanliness, whereas the mainstream home offered this activity.

#### Religious rituals and practices

Family members of residents in mainstream homes described challenges with the resident receiving culturally appropriate personal care. Mainstream homes lacked sufficient staffing resources (both in available staff and staff knowledge and education) to assist with prayer and washing and bathing rituals, which are important for Muslim residents. Staff in the culturally specific homes did not remark on issues relating to available staffing resources, and instead indicated that they understood the importance of cleanliness and did not have to be prompted to carry out specific tasks or assist the resident to carry out religious or cultural rituals. Family members in mainstream homes recounted reminding staff to dress residents with their preferred clothing, such as head scarves for Muslim women; most staff in mainstream homes were not from the same religious or cultural backgrounds and had limited knowledge or training on these practices.

He never missed his prayers, we pray five times a day, because we are Muslim…there’s no facility to be able to do the ritual washing that we have to do before each prayer…there’s no such facility available [in the home] for Muslim people when we do this. (Family, mainstream home)

Cultural norms and beliefs influenced residents’ expectations and experience receiving assistance with personal care. Family and staff in both mainstream and culturally specific homes described challenges providing personal care to opposite sex residents. Residents did not want to receive care from a different sex staff member. Staff in both types of homes struggled to accommodate these requests for male residents because most staff were female. A family member from a mainstream home said that the “care of my mother by men was rather traumatic…. The facility said they will try to minimize that, but of course they were not successful. I don’t think they tried hard enough, but I can understand.” Another family member explained that in their culture, there has always been a gender separation. Men had to be served their meals first by the women, so the idea of a male caregiver was very different and unsettling.

#### Caring relationships

Staff in both culturally specific and mainstream homes suggested that family members and residents from racial and ethnocultural backgrounds had different understandings of the care environment and the relationships between residents and staff compared with White, non-immigrant residents. They believed that cultural differences caused family and residents to have a different understanding and expectation of the care provider–resident relationship in SL homes. For example, staff that cared for South Asian residents described how these older adults often had experience with servants and privately paid care attendants, and they felt that this dynamic manifested in the home.

Even like in India, we can hire a servant for caretaking because they don’t have [this] kind of facility ...That’s the thinking of these people here too…the healthcare aide is [a] kind of servant like they hired back home.” (Staff, culturally specific home)

#### Assimilation

Family members with relatives living in mainstream homes felt their efforts advocating for more culturally sensitive care were ineffective because they were the minority. They described being the only family from that racial or cultural group in the home and as a result their only choice was to tolerate the environment. They felt that because there were not a lot of residents from their cultural or ethnic group or who spoke their language, the facility staff were less willing to adapt to accommodate their needs implicitly requiring that the resident assimilate to the facility rather than the facility accommodate resident differences.

Family members from mainstream and culturally specific homes and residents from culturally specific homes described how the transition to SL was often made easier if the resident had been residing in Canada over a number of years, referring to how long they had been in the country, their English language proficiency, and their level of education. English language ability and familiarity with Canadian food had the greatest impact. One resident indicated that his experience as an immigrant made moving to a care setting like SL easier because he had experienced navigating a new culture and accepting an unfamiliar environment. “I think I have much easier than other person because I do have experience…. You have to accept these challenges, you have to get used to it.” (Resident, culturally specific home).

## Discussion

We interviewed 21 participants including 2 residents, 8 family, and 11 staff from mainstream and culturally specific SL homes. Our primary goal was to examine the perspectives of members of Canada’s largest immigrant groups (South Asian, Chinese, Arab) on access to appropriate care and services in SL homes. In mainstream homes, residents lose the ability to communicate with staff and other residents, eat familiar or religiously sanctioned foods, participate socially, and engage in religious rituals or cultural celebrations. The ability for staff in mainstream homes to provide basic care is hindered by language barriers. Access to culturally familiar food and activities for residents in the culturally specific homes helps the transition to SL and mitigated the guilt and shame reported by family members when placing residents in mainstream homes. Our findings signal the need for further examination of the ethnocultural minority experience across the continuum of publicly funded care (i.e., home care, SL), particularly as it pertains to what contributes to the decision to move or not move to an SL facility.

### Minority ethnic groups encounter challenges and wait times when deciding to move to SL care

Family members described considerable efforts to keep their relative at home or in the community, and when their relative eventually moved to SL, they felt shame and disappointment. Family caregivers from minority ethnic groups experience a complicated mix of emotions related to their role including love, filial responsibility and religious duty, and burnout [34]. Culturally engrained beliefs around the expectation and duty to provide care are pervasive and may contribute to the distress expressed by our family member participants [35].

The long wait times for spaces available in culturally specific homes have negative health implications for older adults, including deteriorating health and physical functioning, and for their caregivers, including increased risk of caregiver burden. In a population-based study using linked administrative health data, caregivers of recent immigrants on the waitlist for long-term care were unable to continue providing care and expressed feelings of distress, anger, and depression [15].

### Communication and care

Staff, particularly in mainstream facilities, struggled to provide safe and effective care for residents due to language issues. A recent integrative review of research examining people with dementia from culturally and linguistically diverse backgrounds in residential settings (primarily NH), identified the need for a common language between residents and staff [31]. These findings echo ours and reiterate how critical communication is to the proper assessment and provision of care. In our study, SL staff did not have on-site access to translators and instead relied on cobbled together methods such as word and picture boards, translation phone applications, and hand signals to discern resident needs. Staff often required family to translate and mediate resident health concerns, which at its worst is unethical and at its least problematic, and is not always feasible if family are unavailable to translate [9]. This same review identified dementia-specific concerns and found that communication and the provision of culturally congruent care may reduce responsive behaviours [31, 36–38]. Frustration with communication issues could be perceived as agitation and managed with medication. A recent study in Alberta (where this study was conducted) compared quality indicators for residents in SL and NH and found that antipsychotic use was consistently higher in SL residents than NH residents; this increased during the COVID-19 pandemic [30]. The focus of this study was not on the quality of clinical care that racialized and ethnoculturally diverse SL residents received; however, our findings do point to specific areas that warrant further qualitative research in tandem with an examination of available administrative health data. These differences are important to examine because although SL is comparable to NH in many ways, there are important distinctions, particularly related to staffing resources that may be influencing resident care. There are many studies of culturally and linguistically diverse residents in care settings [39–42]; however, they are often focused on NH or the studies do not distinguish SL from NH. Future work is needed to better understand the resources and infrastructure available in SL to determine if or how these facilities can provide quality care to diverse residents.

### Culturally sensitive care and loneliness

Ethnic minority older adults have the highest rates of loneliness [43]. Residents who do not speak the dominant language struggled to participate in social activities and often had no one to talk to until their family visited. Some older adults cope with loneliness or social isolation by engaging in social and recreation activities, connecting with their religion, and establishing relationships with staff [18]. These strategies are ineffective if the resident is unable to converse with anyone in the home and if familiar social activities and religious rituals are unavailable. the tensions our participants expressed about their experiences in mainstream SL homes as a minority group bring to mind the fundamental issues of how communities or institutions navigate interactions with one another. In the case of minority residents entering a mainstream SL facility, family members described at least initially trying to engage staff in an acculturative process, which is a process of change that results from contact between different groups [44]. These acculturative processes can establish a sense of understanding and belonging. However, acculturation requires a dual process where both groups engage in learning processes about everyday practices and beliefs; in mainstream homes there were few efforts to meet the needs of diverse residents or change their practices to respond to these residents’ needs. Residents were unable to access appropriate or familiar food or to participate in a variety of cultural celebrations. Family and staff described instances where residents were not able to partake in religious rituals (e.g., washing before prayer) or maintain social and cultural practices (e.g., wear a head scarf). These issues speak to the pervasive staffing issues in the continuing care sector (e.g., limited number of staff, variable training and education) [45], and a lack of awareness or interest on the facility staff’s part in engaging in an acculturation process and adapting their existing organizational systems. Rather than acculturation, family and residents in mainstream homes felt they must assimilate, disregarding their religious rituals and traditions and embracing the existing culture of the SL facility. The denial of their individual experience and the marginalization of their cultural practices that family described in these mainstream homes are associated with loneliness. Studies of migration and multicultural belonging have found that those who have experienced positive acculturative processes feel less lonely [46]. The challenges that our participants described while ‘assimilating’ to the SL facility are in part due to a failure of mainstream SL facilities to ensure that diverse residents feel they belong through culturally congruent care practices.

Culturally sensitive care fosters belonging and control. A scoping review examining barriers to accessing primary care by Canadian immigrants found that one of the most pronounced barriers was related to the gender of the care provider; many people from Asian, South Asian, and Arab backgrounds preferred care providers of their same gender [8]. This is more challenging for male residents because women make up most care providers in continuing care settings. These examples show the pervasive loss of control experienced by minority residents in institutional settings. In general, moving to SL from the community means some degree of loss of control. However, these losses are compounded for minority residents, who in addition to the general loss of autonomy and external social networks, they also lose access to their language, social and cultural activities, and religious rituals [10]. In culturally specific homes, residents might not speak the same language as all the staff, but there were other residents or staff available that did speak their same language or dialect and were familiar with the traditions and practices in the facility; this results in more control and a greater sense of understanding.

### Limitations

This study was conducted in one Canadian province (Alberta). We interviewed 21 participants from mainstream and culturally specific SL facilities. We were only able to interview residents from culturally specific homes because of the challenges in recruiting during the COVID-19 pandemic. Because of COVID-19 visitor restrictions, we were unable to interview residents in person and the mainstream facilities we recruited did not have staffing resources at the time to assist residents to set up the Zoom technology. While we did reach informational redundancy with the inclusion of 2 residents in our total sample, we wholeheartedly acknowledge that more research is needed to specifically understand the resident experience, particularly in mainstream SL facilities. This study is only a preliminary step to more fully understanding the scope of needs of minority populations accessing SL services.

## Conclusion

Many people, regardless of cultural background, are unaware of the continuing care system before they need it. However, minority and immigrant ethnic groups experience more access barriers and lack of information about what support services are available. Residents in mainstream homes cannot easily communicate with staff because of language barriers, leading to loneliness and poor quality of care. In contrast, residents in culturally specific homes had less need to adapt to and navigate new cultural practices. The ease with which residents could understand the cultural activities, food, and practices was one of the main reasons family and residents opted for these homes. Creating standalone facilities for every cultural group is not feasible; therefore, we must improve the care in mainstream facilities, including recruiting more diverse staff and integrating a wider range of recreation and religious services and food options.

### Supplementary Information

**Additional file 1: Supplementary file 1.** Interview Guides.

**Additional file 2: Supplementary file 2.** Interview Data Matrix Template.

## Data Availability

De-identified data may be available in aggregate from the corresponding author on reasonable request.

## Abbreviations

RQI: Rapid qualitative inquiry
SL: Supportive living
