# Supplementary material for: The (un)caring experienced by racialized and/or ethnoculturally diverse residents in supportive living: a qualitative study
Source: BMC Geriatr. 2024 Jan 20;24:78. doi: 10.1186/s12877-023-04636-0 (PMC10800051; doi:10.1186/s12877-023-04636-0)
Supplement: Supplementary file 1 — Additional file 1: Supplementary file 1. Interview Guides. [file 12877_2023_4636_MOESM1_ESM.docx]

Supplementary File 1: Interview Guides

Participant ID: _____

**Family Member Semi-Structured Interview Guide**

This interview concerns the loneliness, social isolation, and care needs of diverse residents in supportive living facilities. The goal of this project is to understand the experience of family members and residents from different racial, religious, and ethno-cultural backgrounds in supportive living facilities. Our work aims to identify potentially unmet needs of residents and contribute to the design of interventions and development of policies in organizations that support care to all residents. There are no right or wrong answers, just try to answer the questions to the best of your knowledge.

First, I am going to ask you about your family member and their move to [name of facility].

1. Tell us about the decision for your loved ones move to [name of facility]
   1. What was the living situation before [resident] moved to the facility?
   2. When did your loved one move to [facility]?
   3. What did you expect moving to [facility name] and did it meet your expectations?
2. Now that they live here, what is your relationship with [resident] like?
   1. How often do you visit in-person?
   2. How often would you say you talk on the phone?
3. Many family members help their loved one with different tasks, are there things that you do to help your [resident]?
   1. For example, going shopping, taking him/her to medical appointments outside the home, bringing in necessary items.

Now we want to talk about the different activities and services available here at [facility]

1. Can you tell us about any social activities or recreation programming that your [resident] attends?
   1. Are there any activities or services your [resident] would like to do but the [facility] does not currently have?

Our project is specifically interested in residents’ social connections and if they are experiencing loneliness

1. What is the first thing that comes to mind when I say the word loneliness?
   1. Any other words or feelings that come to mind?
2. Do you think that your [resident] has or is currently experiencing loneliness?
   1. If yes, have you done anything to address it?
   2. Have the staff ever mentioned concerns about loneliness?

Now we would like to talk about cultural or religious activities here at [facility],

1. Do you feel your loved one is able to practice his/her customs and traditions here at [facility]?
   1. Why or why not?
   2. What might need to be done or made available to access more culturally appropriate care?
2. Are there any difficulties or challenges people from your community [insert here] might face when moving to a facility like [facility name]?
   1. Accessing health and social services?
   2. Communicating with staff?
   3. Communicating with other residents?
3. What is your experience with the staff (care aides, nurses, social worker, recreation) in the facility?
   1. Do you feel they provide culturally appropriate care?
4. What might you recommend facilities like [facility name] do to meet the needs of residents from [community]?

Finally, I will end with some demographic information about you and your resident.

1. Can you please check the box that best reflects your gender?

| ■_1_ Female |
| --- |
| ■_2_ Male |
| ■_3_ Other (please specify): ________ |
| ■_4_ Prefer not to say |

1. Please indicate your age by ticking one of the following

| ■_1_ <20 years |
| --- |
| ■_2_ 20-24 years |
| ■_3_ 25-29 years |
| ■_4_ 30-34 years |
| ■_5_ 35-39 years |
| ■_6_ 40-44 years |
| ■_7_ 45-49 years |
| ■_8_ 50-54 years |
| ■_9_ 55-59 years |
| ■_10_ 60-64 years |
| ■_11_ 65-70 years |
| ■_12_ >70 years |

1. What is your current marital/partner status?

| ■_1_ Single, never married or never lived with a partner |
| --- |
| ■_2_ Married/living with a partner in a common-law relationship |
| ■_3_ Widowed |
| ■_4_ Divorced |
| ■_5_ Separated |
| ■_6_ Prefer not to answer |

1. What is the highest level of education that you have completed?

| ■_1_ Grade school |
| --- |
| ■_2_ High school |
| ■_3_ College or apprenticeship |
| ■_4_ University degree |
| ■_5_ Other (please specify:____________) |

1. In what country were you born?

[If Question 14 is not Canada]

1. In what year did you first come to Canada to live?
2. What was your immigration stream? (e.g., family sponsorship, skilled worker program, skilled trades program, caregiver, refugee and humanitarian resettlement)
3. To which ethnic or cultural groups did your ancestors belong?
4. In what languages can you conduct a conversation?
5. What language do you speak most often at home?
6. What, if any, is your religion?

These next questions are about your (resident) who currently lives in an SL facility

1. What is the resident’s age?
2. Were they born in the same country as you?
   1. If No, specify the country of birth.

[If Question 23 is not Canada]

1. In what year did she/he (resident) first come to Canada to live?
2. What was his/her (resident’s) immigration stream?
3. Does the resident belong to the same ethnic or cultural groups as you?
   1. If No, specify the ethnic or cultural groups.
4. In what languages can he/her (resident) conduct a conversation?
5. What, if any, is his/her (resident’s) religion?

Participant ID: _____

**Resident Semi-Structured Interview Guide**

This interview concerns the loneliness, social isolation, and care needs of diverse residents in supportive living facilities. The goal of this project is to understand the experience of residents, family members, and staff from different racial, religious, and ethno-cultural backgrounds in supportive living facilities. Our work aims to identify potentially unmet needs of residents and contribute to the design of interventions and development of policies in organizations that support care to all residents. There are no right or wrong answers, just try to answer the questions to the best of your knowledge.

First, I am going to ask you about your decision to move to [name of facility].

1. Tell us about the decision to move to [name of facility]
   1. What was the living situation before you moved here?
   2. How long have you lived here?
   3. What did you expect moving to [facility name] and did it meet your expectations?
   4. Do you feel you had a choice in moving to [facility name]?
   5. How does you family feel about your move?
2. Now that they live here, what is your relationship with your family members like?
   1. Would you say you have family or friends that you are close to?
   2. Who do you talk to when you have any problems or concerns?
   3. Who do you talk to if you feel sad or something is upsetting you?
3. How often would you say you see family or friends in person?
4. How often do you talk to them on the phone and/or using the computer?
5. Do you find it hard to stay connected to your family/friends/community?

Our project is specifically interested in residents’ social connections and if they are experiencing loneliness

1. What is the first thing that comes to mind when I say the word loneliness?
   1. Any other words or feelings that come to mind?
   2. Do you ever feel lonely?

Now we want to talk about your life here at [facility name]?

1. How do you feel about your life here?
   1. Is are there things you enjoy about living here?
   2. Are there things that you would change?
2. Do you participate in any social or recreation activities?
   1. Are there any activities or other services you would like but the facility does not have?

Now we would like to talk about cultural or religious activities here at [facility],

1. Do you feel you are able to practice your customs and traditions here at [facility]?
   1. Why or why not?
   2. What might need to be done or made available to access more culturally appropriate care?
2. Are there any difficulties or challenges people from your community [insert here] might face when moving to a facility like [facility name]?
   1. Accessing health and social services?
   2. Communicating with staff?
   3. Communicating with other residents?
3. What is your experience with the staff (care aides, nurses, social worker, recreation) in the facility?
   1. Do you feel they provide culturally appropriate care?
4. What might you recommend facilities like [facility name] do to meet the needs of residents from [community]?

Finally, I will end with some demographic information about you

1. Can you please check the box that best reflects your gender?

| ■_1_ Female |
| --- |
| ■_2_ Male |
| ■_3_ Other (please specify): ________ |
| ■_4_ Prefer not to say |

1. Please indicate your age by ticking one of the following

| ■_1_ <20 years |
| --- |
| ■_2_ 20-24 years |
| ■_3_ 25-29 years |
| ■_4_ 30-34 years |
| ■_5_ 35-39 years |
| ■_6_ 40-44 years |
| ■_7_ 45-49 years |
| ■_8_ 50-54 years |
| ■_9_ 55-59 years |
| ■_10_ 60-64 years |
| ■_11_ 65-70 years |
| ■_12_ 70-74 years |
| ■_13_ 75-79 years |
| ■_14_ 80-84 years |
| ■_15_ 85-89 years |
| ■_16_ 90+ years |

1. What is your current marital/partner status?

| ■_1_ Single, never married or never lived with a partner |
| --- |
| ■_2_ Married/living with a partner in a common-law relationship |
| ■_3_ Widowed |
| ■_4_ Divorced |
| ■_5_ Separated |
| ■_6_ Prefer not to answer |

1. In what country were you born?

[If Question 14 is not Canada]

1. In what year did you first come to Canada to live?
2. What was your immigration stream? (e.g., family sponsorship, skilled worker program, skilled trades program, caregiver, refugee and humanitarian resettlement)
3. To which ethnic or cultural groups did your ancestors belong?
4. What, if any, is your religion?

Participant ID: _____

**Direct Care Staff Semi-Structured Interview Guide**

This interview concerns the loneliness, social isolation, and care needs of diverse residents in supportive living facilities. The goal of this project is to understand the experience of staff, family members and residents from different racial, religious, and ethno-cultural backgrounds in supportive living facilities. Our work aims to identify potentially unmet needs of residents and contribute to the design of interventions and development of policies in organizations that support care to all residents. There are no right or wrong answers, just try to answer the questions to the best of your knowledge.

First, I am going to ask you about your position here at [facility name]?

1. What is your position?
   1. How long has you worked here at [facility name]?

Our project is interested in understanding the experience of residents who are from diverse backgrounds, many of whom do not speak English as their first language and who come from different religious and cultural backgrounds.

1. We are going to talk about your experience providing care to diverse residents in SL.
   1. What proportion of residents in this facility would you think are from these diverse backgrounds?
   2. In general, what has been your experience caring for diverse residents?
      1. Challenges, benefits
   3. What is your experience interacting with the family of these residents?
2. Have you cared for residents that do not speak English? What were some of the differences caring for these residents compared to those that do speak English?
   1. Communicating with staff
   2. Communicating with other residents
3. Different religious and cultural groups have different traditions and practice related to food, for example, those who are Muslim may eat Halal (only eating meat that adheres to a specific dietary standard), people from the Jewish faith may keep kosher (food prepared in adherence with Jewish dietary laws).
   1. Do you know of any residents that adhere to these food practices?
   2. Do you understand the significance of these practices? Were any specific food practices like these provided for any of your residents? Is information like this available in the resident care plan?
   3. In your training as a [position], did you talk about caring for residents from different religious or cultural groups?
   4. Have you ever talked to other colleagues or management about the differences caring for diverse residents?
4. Our project looks at resident loneliness and isolation, particularly for those that are from diverse backgrounds, including those from different cultures and who may not speak English. What has been your experience with diverse residents attending various recreation activities?
   1. Probe: not attending because they do not speak English, staying in their room, rely on family for socialization
5. Do you think these residents are lonely, especially those that do not speak English?
   1. Have you ever experienced challenges detecting loneliness for residents who don’t speak English?
   2. Are there any physical or verbal cues that might alert you to a resident’s loneliness?
   3. Do you have ways to document if a resident is lonely?
6. What might you recommend facilities like [facility name] do to meet the needs of residents from different ethno-cultural or religious backgrounds?

Finally, I will end with some demographic information about you

1. Can you please check the box that best reflects your gender?

| ■_1_ Female |
| --- |
| ■_2_ Male |
| ■_3_ Other (please specify): ________ |
| ■_4_ Prefer not to say |

1. Please indicate your age by ticking one of the following

| ■_1_ <20 years |
| --- |
| ■_2_ 20-24 years |
| ■_3_ 25-29 years |
| ■_4_ 30-34 years |
| ■_5_ 35-39 years |
| ■_6_ 40-44 years |
| ■_7_ 45-49 years |
| ■_8_ 50-54 years |
| ■_9_ 55-59 years |
| ■_10_ 60-64 years |
| ■_11_ 65-70 years |
| ■_12_ >70 years |

1. What is your current marital/partner status?

| ■_1_ Single, never married or never lived with a partner |
| --- |
| ■_2_ Married/living with a partner in a common-law relationship |
| ■_3_ Widowed |
| ■_4_ Divorced |
| ■_5_ Separated |
| ■_6_ Prefer not to answer |

1. What is the highest level of education you have completed?

| ■_1_ Grade school |
| --- |
| ■_2_ High school |
| ■_3_ College or apprenticeship |
| ■_4_ University degree |
| ■_5_ Other (please specify:____________) |

1. In what country were you born?

[If Question 14 is not Canada]

1. In what year did you first come to Canada to live?
2. What was your immigration stream? (e.g., family sponsorship, skilled worker program, skilled trades program, caregiver, refugee and humanitarian resettlement)
3. To which ethnic or cultural groups did your ancestors belong?
4. In what languages can you conduct a conversation?
5. What language do you speak most often at home?
6. What, if any, is your religion?

Participant ID: _____

**Manager Semi-Structured Interview Guide**

This interview concerns the loneliness, social isolation, and care needs of diverse residents in supportive living facilities. The goal of this project is to understand the experience of staff, family members and residents from different racial, religious, and ethno-cultural backgrounds in supportive living facilities. Our work aims to identify potentially unmet needs of residents and contribute to the design of interventions and development of policies in organizations that support care to all residents. There are no right or wrong answers, just try to answer the questions to the best of your knowledge.

First, I am going to ask you about your position here at [facility name]?

1. What is your position?
   1. How long has you worked here at [facility name]?
   2. How long have you worked in supportive living or long-term care facilities overall?

Our project is interested in understanding the experience of residents who are from diverse backgrounds, many of whom do not speak English as their first language and who come from different religious and cultural backgrounds.

1. What proportion of residents in this facility would you think identify as having a background that differs either in terms of language, religion, or other cultural believes from the majority of the facility’s residents?
   1. Do you have any formal way of tracking this type of resident information (e.g., ethnicity, languages spoken, religion)?
   2. Is any of this type of information collected on admission?
   3. Is any of this type of information collected or tracked after admission?
   4. If so, how is it communicated to the staff who provide care for these particular residents?

**Food**

1. Different religious and cultural groups have different traditions and practice related to food, for example, those who are Muslim may eat Halal (only eating meat that adheres to a specific dietary standard), people from the Jewish faith may keep kosher (food prepared in adherence with Jewish dietary laws).
   1. Is the meal planning and service at the facility inclusive of cultural dietary regulations and preferences? How is it managed or addressed within the facility?
   2. How are resident dietary regulations and preferences documented?
   3. Do you understand the significance of these practices? Were any specific food practices like these provided for any of your residents? Is information like this available in the resident care plan?
   4. If such dietary practices could not be accommodated, could you talk a little bit about why this could not be done? What barriers do you think prevented dietary preferences or practices from being honoured?

**Religious rituals, practices, and preferences**

1. Some residents prefer to receive care from a member of the same sex, this can be for a variety of reasons, some of which are religious and cultural. Are there systems in place at [name of facility] to enable residents to receive care from the same sex?
2. Is there space in the facility for prayer? Accommodations to assist with prayer practices.
3. Is there an opportunity for a variety of social programs to reflect cultural and/or religious diversity? (e.g., holiday celebrations)

**Organizational practices**

1. Does your organization (if a part of a chain) or facility require any specific training or education related to equity, diversity, or inclusion for either staff about meeting resident needs?
2. Can you speak to any instances of conflict between residents, staff, or family members that was related to differences in cultural or language differences?
   1. Is there a process to deal with intercultural conflict between staff, family, and residents?
3. Is there a process to identify and address resident and family concerns related to equity and diversity?
   1. Ensure services are inclusive?
   2. Availability of language and/or religious services/activities?
4. What resources would your organization need that it currently does not have to address some of the systemic barriers that prevent you from meeting residents’ religious, cultural, or language needs? (e.g., educator/facilitator, access to materials)

Finally, I will end with some demographic information about you

1. Can you please check the box that best reflects your gender?

| ■_1_ Female |
| --- |
| ■_2_ Male |
| ■_3_ Other (please specify): ________ |
| ■_4_ Prefer not to say |

1. Please indicate your age by ticking one of the following

| ■_1_ <20 years |
| --- |
| ■_2_ 20-24 years |
| ■_3_ 25-29 years |
| ■_4_ 30-34 years |
| ■_5_ 35-39 years |
| ■_6_ 40-44 years |
| ■_7_ 45-49 years |
| ■_8_ 50-54 years |
| ■_9_ 55-59 years |
| ■_10_ 60-64 years |
| ■_11_ 65-70 years |
| ■_12_ >70 years |

1. What is your current marital/partner status?

| ■_1_ Single, never married or never lived with a partner |
| --- |
| ■_2_ Married/living with a partner in a common-law relationship |
| ■_3_ Widowed |
| ■_4_ Divorced |
| ■_5_ Separated |
| ■_6_ Prefer not to answer |

1. What is the highest level of education you have completed?

| ■_1_ Grade school |
| --- |
| ■_2_ High school |
| ■_3_ College or apprenticeship |
| ■_4_ University degree |
| ■_5_ Other (please specify:____________) |

1. In what country were you born?

[If Question 15 is not Canada]

1. In what year did you first come to Canada to live?
2. What was your immigration stream? (e.g., family sponsorship, skilled worker program, skilled trades program, caregiver, refugee and humanitarian resettlement)
3. To which ethnic or cultural groups did your ancestors belong?
4. In what languages can you conduct a conversation?
5. What language do you speak most often at home?
6. What, if any, is your religion?
