# Supplementary material for: The (un)caring experienced by racialized and/or ethnoculturally diverse residents in supportive living: a qualitative study
Source: BMC Geriatr. 2024 Jan 20;24:78. doi: 10.1186/s12877-023-04636-0 (PMC10800051; doi:10.1186/s12877-023-04636-0)
Supplement: Supplementary file 2 — Additional file 2: Supplementary file 2. Interview Data Matrix Template. [file 12877_2023_4636_MOESM2_ESM.docx]

Supplementary File 2: Interview Data Matrix Template

|  |  | **Transition to SL** | | **Activities** | | **Services** | | **Language** | | **Organizational Practices** |
| --- | --- | --- | --- | --- | --- | --- | --- | --- | --- | --- |
| **Participant ID** | **Facility Type** | **Decision to move** | **Choosing a facility** | **Recreation and Social** | **Religious and Cultural** | **Food** | **Medical and Healthcare** | **Barriers and Facilitators** | **Resources** | **Education and Resources** |
| 1 |  |  |  |  |  |  |  |  |  |  |
| 2 |  |  |  |  |  |  |  |  |  |  |
| 3 |  |  |  |  |  |  |  |  |  |  |
| 4 |  |  |  |  |  |  |  |  |  |  |
